# The Prevalence of Depression and Anxiety in Heart Failure Patients in Saudi Arabia: An Original Study

**DOI:** 10.7759/cureus.36997

**Published:** 2023-04-01

**Authors:** Mostafa Q Al Shamiri, Abdullah A Almushawah, Abdulaziz H Alsomali, Mishal B Alsuwayegh, Mohammed A Aljaffer, Ahmad M Hayajneh, Priyadarshi Prajjwal

**Affiliations:** 1 Cardiology, King Saud University, Riyadh, SAU; 2 Psychiatry, King Saud University, Riyadh, SAU; 3 Neurology, Bharati Vidyapeeth University Medical College, Pune, IND

**Keywords:** comorbidities, heart failure, cardiovascular disease, anxiety, depression

## Abstract

Background

Depression is a severe comorbidity that is often detected in patients with chronic diseases. Poor prognosis may eventuate high mortality risk. Up to 30% of heart failure patients have been documented with depression and the majority upholds depression-related symptoms that may have serious clinical implications, such as hospital readmissions and fatalities. To mitigate depression-induced harms among heart failure patients, studies are being conducted to determine the prevalence, risk factors, and interventions.

Objectives

The current investigation is envisioned to examine the prevalence of depression and anxiety among the Saudi heart failure population. Also, it will help to explore the risk factors that will subsequently facilitate the analysis of preventive measures.

Methodology

The cross-sectional epidemiologic research was conducted at King Khalid University, Hospital with the recruitment of 205 participants. Each participant underwent a 30-question screening for depression, anxiety, and related risk factors. The “Hospital, Anxiety, and Depression Scale” (HADS score) was used to score subjects for the assessment of both comorbidities. The data points were subsequently analyzed by descriptive statistics and regression analysis.

Results

Among 205 participants, 137 (66.82%) were male and 68 (33.17%) were female with a mean age of 59.71 years. Our sample reflects a prevalence of 52.7% depression and 56.9% anxiety in Saudi heart failure patients. High depression scores were positively related to age, female gender, hospital readmissions, and pre-existing comorbidities in heart failure patients.

Conclusion

The study manifested high depression scores among the Saudi heart failure cohort compared to the previous survey. In addition, a substantial interrelationship of depression and categorical variables has been identified that accentuates predominating risks that can potentially promote depression and anxiety in heart failure patients.

## Introduction

Heart failure (HF) is a chronic impairment of cardiac function leading to inefficient pumping of blood and oxygen. Typical symptoms include dyspnea, fatigue, and/or edema [1]. Globally, 224.1 million people are afflicted with the disease accounting for a death toll of 17.9 million each year which constitutes 32% of overall mortalities [2,3]. The condition is often associated with a multitude of comorbidities that affect the quality of life (QOL) of the patients. Some major public health concerns in HF patients include depression and anxiety, which can potentially influence all life aspects of the patients. Up to 30% of HF patients have been reported with depression and to a greater extent suffer from depression-related symptoms that may translate into severe clinical outcomes, particularly rehospitalizations, and mortalities. Recent estimates have anticipated a dramatic increase in comorbid depression and HF in the upcoming years [4,5]. Several studies have established different factors that may induce depression among HF patients. These may include smoking, lack of social support, inadequate self-care behavior, age, previous episodes of depression, and females being more likely to be comorbid with depression [6]. These mutual associations together play a role in leading to the development of depression in this cohort. Prevalence rates in Saudi Arabia of these relatable diseases were measured about two decades ago [7], which signifies the quantification of the latest figures and determination of the depression-leading factors among Saudi HF patients. Correspondingly, we aim to examine the prevalence and risk factors of depression and anxiety among Saudi HF patients and suggest preventive measures to mitigate them.

## Materials and methods

Methodology

Responses were collected through a web-based and self-administered questionnaire. Non-probability sampling technique was implied for the study. The study commenced after obtaining ethical approval from the Institutional Review Board (IRB) and voluntary participation consent of the HF patients visiting the cardiology clinic of King Khalid University Hospital. Over the course of seven months, 205 patients filled out the questionnaire. Inclusion criteria involve the restricted participation of HF patients. Non-HF and unschooled patients were excluded from the study. All the participants obtained directions on how to fill out the questionnaire. Patients’ depression and anxiety were measured through Hospital Anxiety and Depression Scale (HADS). HADS is a validated and reliable tool to precisely estimate the presence and severity of depression and anxiety. It facilitates the apprehending of the suffering experience and the design of personalized intervention [8]. The calculation of sample size was based on the following considerations: standard normal distribution value at 95% confidence level (z) = 1.96, and margin of error (d) = 5%.

Study instruments

A comprehensive questionnaire was composed (entailing 16 questions) to assess sociodemographic features and sample characteristics including age, sex, marital status, number of spouses, number of children, education level, monthly income, job type, self-score on health, number of medications, sources to acquire medicine, time of HF diagnosis, causes of HF, previous year hospitalizations, exposure to smoking and support at home. The Arabic questionnaire was provided to the participants for the measurement of depression and anxiety-related symptoms. Fourteen items were established exhibiting two subscales, one for depression and the other for anxiety. Scoring was performed on the HADS scale (0-3), where 0 refers to the lowest value and 3 displays the highest severity of anxiety and depression. Final scoring was performed by the summation of subscale values. Results varied from 0 to 21. Score count guided the establishment of three levels namely, normal (0-7), borderline (8-10), and abnormal (11-21).

Statistical analysis

Data were statistically analyzed using SPSS version 27. Descriptive statistics were employed to determine the characteristics of the data set. The mean and student t-tests were used to measure the significant differences (p-value) among categorical values. Frequency distribution rendered cumulative percentages to delineate the cause of HF. Analysis of Variance (ANOVA) appraised the relationship of the dependent variable (anxiety and depression) with the independent variables (sociodemographic factors and HF-linked variables). Data estimation was performed at a 95% Confidence Interval (CI) and p<0.05 was considered significant. The data output was finally presented in the form of tables and charts.

## Results

In total, 205 HF patients contributed to the study. In our study, there were 137 males and 68 females. The majority of study participants were married and had a single wife and 2-3 children. A significant frequency of individuals with bachelor's degrees and high school diplomas was identified. The interviewees were mainly employed by the government with a monthly income of 5,000-10,000 Saudi Riyal. The majority of the candidates submitted below-average scores for their health. Support was primarily provided by spouses or children. In most cases, medicines were issued from the hospital, with an average intake of 4-7 medicines a day per individual. The totality of analyses manifested depression persistence at 52.7% and anxiety at 56.9% (Table 1). Patients that did not suffer from depression or anxiety were estimated to be 47.31%, and 43.4% respectively (Table 1). Collectively, 16.09% of patients moderately experienced depression-related symptoms, whereas 36.58% had critical symptomatology. Comparably, 18.04% of patients exhibited borderline anxiety, while 38.53% reported high suffering from anxiety. The average age of the research sample was 59.71 years, with the youngest responder being 24 years old and the oldest being 95 years old. The highest depression scores were recorded at ages 52 (n=15) and 60 (n=11). An elevated standard deviation indicates high variability of the data point (age). Out of 119 depressed individuals, 73.5% of women and 50.3% of men reported depression in their responses. Comorbidities, such as coronary artery disease (CAD), valvular heart disease (VHD), hypertension, diabetes mellitus (DM), and cardiomyopathy, were reported with depression percentages of 49.4, 87.5, 58.8, 56.7, and 55.7, respectively (Table 2). Of the 117 patients that reported previous year hospitalization, 75 HF patients were spotted with depression and 72 patients reported anxiety. A substantial relationship between depression and sociodemographic factors such as age (95% CI, p: 0.035), gender (95% CI, p: 0.049), and hospital readmissions (95% CI, p: 0.020) was verified by correlational analysis (Table 1).

**Table 1:** Sociodemographic details of the HF patients with relation to depression and anxiety

	Mean	Std Deviation	P-value	Total Cohort, N= 205	Depressed, N (%)	Anxiety, N (%)	Total Depression/Anxiety, N (%)
Age (Mean)	59.71	12.03	0.035	59.71	108 (52.7)	116 (56.9)	224 (100)
Male	0.66	0.47	0.049	137	69 (50.3)	71 (51.8)	140 (62.5)
Female	0.33			68	50 (73.5)	47 (69.1)	97 (43.30)
Number of Children	3.34	0.99	0.29				
None				23	18 (78.2)	16 (69.5)	34 (15.17)
1				8	6 (75)	4 (50)	10 (4.46)
2-3				49	23 (46.9)	26 (53.0)	49 (21.87)
>3				125	72 (57.6)	72 (57.6)	144 (64.28)
Spouse Caregiver	0.50	0.52	0.37	104	47 (45.1)	50 (48.0)	138 (43.30)
Children Caregiver	0.52	0.50	0.59	107	60 (56.07)	61 (57)	116 (54.01)
Siblings Caregiver	0.11	0.32	0.03	24	20 (83.3)	20 (83.3)	197 (17.85)
Driver/Maid Caregiver	0.11	0.32	0.97	24	16 (66.6)	16 (66.6)	205 (14.28)
Smoking	1.55	0.498	0.77	93	49 (52.6)	44 (47.3)	93 (41.51)
Previous Year Hospitalization (PYH)	1.76	0.77	0.02				
0				89	42 (47.1)	44 (49.4)	86 (38.39)
1-2 times				79	51 (64.5)	51 (64.5)	102 (45.53)
>3 times				38	24 (63.1)	21 (55.2)	45 (20.08)

**Table 2:** Causes of heart failure CAD: Coronary Artery Disease, VHD: Valvular Heart Disease, DM: Diabetes Mellitus

	Total Cohort, N= 205	Mean	P-value		Depressed, N (%)	Anxiety, N (%)	Total Depression/Anxiety, N (%)
CAD	87	0.42	0.49	0.06	43 (49.4)	46 (52.8)	89 (39.73)
VHD	16	0.07	0.26	0.45	14 (87.5)	13 (81.2)	27 (12.05)
DM	67	0.32	0.47	0.47	38 (56.7)	41 (61.1)	79 (35.26)
Hypertension	68	0.33	0.47	0.82	40 (58.8)	43 (63.2)	83 (37.05)
Cardiomyopathy	52	0.25	0.53	0.81	29 (55.7)	24 (46.1)	53 (23.66)

## Discussion

Depression is a progressively rising condition in the global population. Current analysis has established a fairly high prevalence of depression in the Saudi-HF cohort. More than half of the Saudi-Arabian HF cohort experienced depression and anxiety. In a similar analysis, recent South African research estimated 52.4% depression and 53.4% anxiety in the HF cohort [9]. A cross-sectional study of Northwest Ethiopia conducted in 2017 produced similar findings, where 51.5% of HF patients experienced depression due to indigent self-care, lack of social support, smoking, female gender, and prolonged HF history [6]. A retrospective analysis of 2007-2016 pointed out 17% depression in USA HF patients, with poor health and poverty serving as the main predictors of comorbidity (Table 3) [10]. In a recent meta-analysis, it was found that 31.3% of global HF patients experience depression, 32.9% suffered anxiety, and 57.7% were stressed [11].

**Table 3:** Comparative multi-country analysis of different variables in patients with heart failure and depression CAD: Coronary Artery Disease, VHD: Valvular Heart Disease

Country	Depression	Age	Gender (P-value)	CAD	VHD	Hypertension	Diabetes	Cardiomyopathy	Hospitalizations/poor health status	References
Saudi Arabia	20% 2004	≥60	0.19	72.2%	Absent	69.4%	58.3%	Absent	27.2%	(Mohsen et. al) [7]
USA	17% 2007-2016	≥60	0.005	Absent	Absent	Absent	Absent	Absent	76.4%	(Chobufo et. al) [10]
Northwest Ethiopia	51.5% 2017	52.3	0.01	Absent	Absent	48.6%	4.2%	Absent	44.9%	(Yazew et. al) [6]
South Africa	52.4% 2021	53	0.16	Absent	Absent	35.9%	Absent	Absent	Absent	(Tsabedze et. al) [9]
Current Analysis	52.7% 2022	59.71	0.049	49.4%	87.5%	56.7%	58.8%	55.7%	64.5%	

The high depression and stress in current studies can be explained by the fact that mental health is influenced by changes in lifestyle over time. Lack of physical activity, suboptimal sleep, sedentary lifestyle, and more proclivity towards smoking and alcohol consumption have altered mental health and imposed high psychological distress [12]. Chronic co-morbidities may potentially be a major factor in the progression of depression in the HF population. The current study revealed high depression scores in more than half of the HF patients that were afflicted with VHD, hypertension, DM, and cardiomyopathy. About one in every five patients that suffered from coronary artery disease, HF, and peripheral artery disease, were comorbid with depression [13]. In a clinical trial, ischemic heart disease (44%), hyperlipidemia (48%), hypertension (63%), diabetes (33%), atrial fibrillation (25%), and chronic kidney disease (25%) were principally present in depressed HF patients [14]. In coherence, Bahall et al. also signified hypertension, DM, stress in life, and a family history of ischemic heart disease as the commonest risk factors linked to depression in the HF cohort [15].

In 2004, about 20% of Saudi Arabian HF patients were found to be suffering from depression, whereas the previous past five years' estimates rendered 56.7% (2021) [16], 62%-65% (2018) [17], and 23.52% (2019) [18] depression in Saudi HF cohort. Compared to the 2019 report, the current analysis corroborated the increase of 20% signifying an upsurge of the associated risk factors [7]. Researchers are attempting to establish a possible connection for the co-occurrence of these diseases. Attributing to the previous literature, we assessed the sociodemographic features, lifestyle, and medical history of Saudi HF patients. In our analysis, the main risk variables for depression were gender, age, and frequent hospitalizations. Female respondents reported a strikingly high depression score despite being underrepresented in the sample. Comparably, a study of the year 2020 has found a significant gender-based association of HF with depression, where a high incidence is noted among females accompanied by a high fatality rate [19]. With reference to the COVID pandemic, women were diagnosed with chronic and severe adversities including depression and encountered high incidences of HF. Thus, a pronounced interplay is observed between depression and female-associated HF. Underlying mechanisms presumably involve hyper-activeness, sympathetic nervous system, and dysfunction of hypothalamus-pituitary-adrenal glands [12]. In coherence with our investigation, a study has established an independent association between age and major depressive disorder among patients with heart diseases posing severe psychological distress [20]. Likewise, some observational, prospective, and comparative studies underscored the high prevalence of depression among elderly-chronic HF patients ensued by isolated systolic hypertension and lack of social support. Pathophysiological mechanism encompasses complex reciprocity of behavioral factors, neurohormonal activation, and inflammation that subsequently leads to vascular damage and hypercoagulation resulting in chronic HF [21]. Our analysis also manifested a significant association between increased hospitalizations that can potentially elevate depression in HF patients. This is presumptively connected with poor or fragile health conditions. Hence, it demonstrates the direct proportionality of depression and impaired heart conditions. In accordance, 25%-50% of depression and 10%-50% of anxiety were recorded in patients with chronic heart disease [22]. Besides, a dose-response relationship of depression was observed in patients with cardiac diseases. The risk of an adverse cardiac event was recorded 1.96 times in patients with moderate depression, which is higher than 2.81 times from the patients with no symptomatology of depression [22]. Based on the current evidence, a positive correlation between depression and HF can be established. The severity of HF may promote depression. Management of mild to moderate depression can be performed by implying early screening, cognitive behavioral therapy, and exercise. The severity of the disease can be additionally supported by antipsychotic drugs. High-risk depression/anxiety may require immediate psychiatric care and brain simulations.

Limitations

There were some limitations in our original study which should be mentioned and documented. Firstly, there is not enough research on the association of depression and anxiety with HF. Also, there were many unschooled participants with difficulty understanding the questionnaire, so we excluded them resulting in a decreased sample size. Extensive data analysis and several studies are required to fully comprehend this major global burden of the co-existence of both diseases.

## Conclusions

Depression is an emergent and severe issue that accompanies several chronic diseases, including HF. Over 50% Saudi HF cohort exhibited depressive symptoms. In addition, various sociodemographic features and clinical traits are associated with high associations of HF with depression. To corroborate the further understanding of the prevalence of both clinical conditions together, extensive sampling and data analysis are required.
